# Metabolic flux analysis and the NAD(P)H/NAD(P)^+^ ratios in chemostat cultures of *Azotobacter vinelandii*

**DOI:** 10.1186/s12934-018-0860-8

**Published:** 2018-01-22

**Authors:** Andres García, Pau Ferrer, Joan Albiol, Tania Castillo, Daniel Segura, Carlos Peña

**Affiliations:** 1 Departamento de Ingeniería Celular y Biocatálisis, Instituto de Biotecnología, Universidad Nacional Autónoma de México, Av. Universidad 2001, Col. Chamilpa Cuernavaca, Apdo. Post. 510-3, 62210 Cuernavaca, Morelos Mexico; 2 Departament d’Engiyeria Química, Biològica i Ambiental, Escola d’Enginyeria, Universitat Autònoma de Barcelona, Bellaterra (Cerdanyola del Vallés), Barcelona, Spain; 3 Departamento de Microbiología Molecular, Instituto de Biotecnología, Universidad Nacional Autónoma de México, Av. Universidad 2001, Col. Chamilpa Cuernavaca, 62210 Cuernavaca, Morelos Mexico

**Keywords:** NAD(P)H/NAD(P)^+^ ratios, Metabolic flux analysis, Oxygen availability, *Azotobacter vinelandii*

## Abstract

**Background:**

*Azotobacter vinelandii* is a bacterium that produces alginate and polyhydroxybutyrate (P3HB); however, the role of NAD(P)H/NAD(P)^+^ ratios on the metabolic fluxes through biosynthesis pathways of these biopolymers remains unknown. The aim of this study was to evaluate the NAD(P)H/NAD(P)^**+**^ ratios and the metabolic fluxes involved in alginate and P3HB biosynthesis, under oxygen-limiting and non-limiting oxygen conditions.

**Results:**

The results reveal that changes in the oxygen availability have an important effect on the metabolic fluxes and intracellular NADPH/NADP^+^ ratio, showing that at the lowest OTR (2.4 mmol L^−1^ h^−1^), the flux through the tricarboxylic acid (TCA) cycle decreased 27.6-fold, but the flux through the P3HB biosynthesis increased 6.6-fold in contrast to the cultures without oxygen limitation (OTR = 14.6 mmol L^−1^ h^−1^). This was consistent with the increase in the level of transcription of *phbB* and the P3HB biosynthesis. In addition, under conditions without oxygen limitation, there was an increase in the carbon uptake rate (twofold), as well as in the flux through the pentose phosphate (PP) pathway (4.8-fold), compared to the condition of 2.4 mmol L^−1^ h^−1^. At the highest OTR condition, a decrease in the NADPH/NADP^+^ ratio of threefold was observed, probably as a response to the high respiration rate induced by the respiratory protection of the nitrogenase under diazotrophic conditions, correlating with a high expression of the uncoupled respiratory chain genes (*ndhII* and *cydA*) and induction of the expression of the genes encoding the nitrogenase complex (*nifH*).

**Conclusions:**

We have demonstrated that changes in oxygen availability affect the internal redox state of the cell and carbon metabolic fluxes. This also has a strong impact on the TCA cycle and PP pathway as well as on alginate and P3HB biosynthetic fluxes.

## Background

*Azotobacter vinelandii* is a Gram negative bacterium that produces two polymers of biotechnological importance, poly-3-hydroxybutyrate (P3HB), an intracellular polyester of the polyhydroxyalkanoates (PHAs) family, and alginate, an extracellular polysaccharide [1, 2]. This bacterium fixes nitrogen under aerobic conditions, being able to protect its oxygen sensitive nitrogenase complex from damage by respiratory protection [3]. Due to this characteristic, *A. vinelandii* exhibits a high respiratory activity, especially when exposed to high oxygen concentrations [4]. Therefore, when it is grown without control of the dissolved oxygen tension (DOT), the cultures operate at a DOT close to zero (microaerophilic conditions). Under this condition, the maximum oxygen transfer rate (OTR_max_) and/or the maximum oxygen uptake rate (OUR_max_) have been used to evaluate the respiratory metabolism of *A. vinelandii* [5, 6]. Several studies been focused on how the agitation rate and the OTR_max_ affect alginate and P3HB production [7–10], showing that the OTR_max_ is positively correlated with alginate biosynthesis, whereas P3HB biosynthesis follows an inverse relation.

Several studies have reported the relationship between the NAD(P)H^+^/NAD(P) cofactor levels and the metabolic flux distributions in aerobic cultures. For example, genetic manipulations aiming at perturbing NADH cofactor levels and/or regeneration rates were employed as a tool for the metabolic engineering of *Escherichia coli*  [11–13], *Lactococcus lactis*  [14], *Bacillus subtilis* [15, 16] and *Saccharomyces cerevisiae*  [17]. These studies showed that changes in the ratio of NADH/NAD^+^ determined the metabolic products. Other studies have shown that strategies increasing the NADPH cofactor levels improved, for example, penicillin formation [18], methylenomycin biosynthesis [19] and P3HB biosynthesis [20–23].

To our knowledge, there have been no studies about the relationship between NADH and NADPH levels and the metabolic behavior of *A. vinelandii*. However, it is well known that the NADPH is consumed during PHB biosynthesis and the alginate biosynthesis produces NADH in this bacterium. For example, the limiting step and the control point for alginate biosynthesis is the activity of the GDP-mannose dehydrogenase enzyme, which is involved in the irreversible oxidation of GDP-mannose to GDP-mannuronic acid [24], and is NAD^+^-dependent [25]. This enzyme performs a double oxidation, in which two molecules of NADH are released for each GDP-mannuronic acid synthesized; therefore, the biosynthesis of alginate could be affected by changes in the intracellular redox state. So far, there is no evidence pointing at a possible effect of the reduction power on the production of this polymer. On the other hand, the accumulation of reducing power (NADH and NADPH) in cells growing under oxygen-limiting conditions is probably involved in the allosteric down-regulation of some TCA cycle enzymes such as citrate synthase and isocitrate dehydrogenase, decreasing the flux of acetyl-CoA into this cycle, thereby increasing its availability for P3HB biosynthesis [26]. This is because a molecule of NADHP being required for each monomer of 3-hydroxybutyryl formed during P3HB biosynthesis.

Recently, in *A. vinelandii* growing in shake flasks it was demonstrated through metabolic flux analysis [27] that changes in oxygen availability have an important impact on the metabolic fluxes, which was also reflected on the alginate and P3HB yields. However, the NADH/NAD^**+**^ and NADPH/NADP^**+**^ ratios were not quantified, therefore preventing dissection of the interplay between the rate of redox cofactor regeneration, the intracellular carbon flux distribution and the rate of carbon substrate uptake. In the present study, the relationship of the intracellular NAD(P)H/NAD(P)^**+**^ ratios and metabolic flux analysis through the central carbon metabolism were analyzed in *A. vinelandii* cultures grown under oxygen-limiting and non-limiting conditions.

## Results

### DOT and OTR under oxygen-limiting and non-limiting conditions

**Fig. 1:**
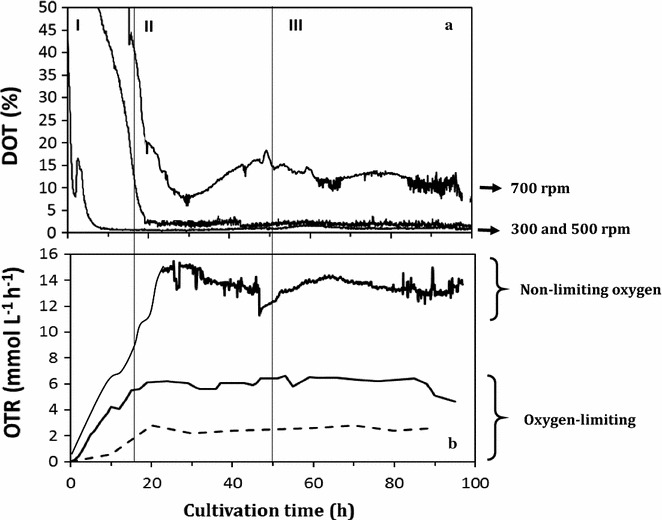
DOT profiles (**a**) and OTR (**b**) in *A. vinelandii* ATCC 9046 grown in continuous cultures at 300, 500 and 700 rpm. (I) Batch stage, (II) continuous state, (III) steady-state

The main purpose of this study was to understand the effect of the oxygen transfer rate (OTR) on the distribution of metabolic fluxes and the intracellular redox state (i.e. NADH/NAD^+^ and NADPH/NADP^+^ ratios) in *A. vinelandii* growing under oxygen-limiting and non-limiting conditions. The dissolved oxygen tension (DOT) (a) and OTR (b) at 300, 500 and 700 rpm agitation rates in continuous cultivations at a dilution rate of 0.08 h^−1^ are shown in Fig. 1. Due to the high oxygen consumption rate of *A. vinelandii*, under oxygen-limiting conditions (300 and 500 rpm), DOT levels remained close to zero during the steady-state growth (Fig. 1a). For the cultures conducted at 700 rpm, the average DOT was 11.4 ± 2.3% during the steady-state (between 50 and 96 h of cultivation). These results show that the cultures performed at 300 and 500 rpm resulted to be oxygen-limiting, whereas the cultures run at 700 rpm were not oxygen-limited.

The effect of agitation rate on the OTR profiles of chemostat cultures is shown in Fig. 1b. During the steady-state there is no oxygen accumulation (dO_2_/dt ≈ 0); therefore, the OTR is equal to the oxygen uptake rate (OUR). For all tested conditions, a maximum OTR value was reached at the steady-state, representing the maximum gas–liquid mass transfer capacity of the bioreactor system [28]. Specifically, the OTRs reached maximum values of 2.4 ± 0.03, 6.2 ± 0.05 and 14.3 ± 0.3 mmol L^−1^ h^−1^ for 300, 500 and 700 rpm, respectively. This had an important effect on the specific oxygen uptake rate (*qO*_*2*_) (Table 1), which was up to 4.7-fold higher for the highest OTR condition with respect to that of the lowest OTR.

**Table 1:** Kinetic parameters during steady-state of *A. vinelandii* ATCC 9046, grown in continuous cultures (*D* = 0.08 h^−1^) under oxygen-limiting (300, 500 rpm) and non-limiting oxygen (700 rpm) conditions

Agitation rate (rpm)	OTR_max_ (mmol L^−1^ h^−1^)	Y_protein/glucose_ (g g^−1^)	qO_2_ (mmol g_prot_^−1^ h^−1^)	Alginate production		P3HB production	
				(g L^−1^)	Y_Alg/prot_	(g L^−1^)	Y_PHB/prot_
300	2.4 ± 0.32	0.15 ± 0.02	3.7 ± 0.16	0.7 ± 0.08	1.01	0.75 ± 0.04	1.15
500	6.2 ± 0.57	0.11 ± 0.01	8.26 ± 0.24	1.18 ± 0.18	1.52	0.34 ± 0.05	0.43
700	14.3 ± 1.2	0.08 ± 0.02	20 ± 0.34	1.07 ± 0.15	1.50	0.05 ± 0.002	0.07

The biomass (X) corresponds to the residual

### Glucose consumption, biomass, alginate and P3HB production

**Fig. 2:**
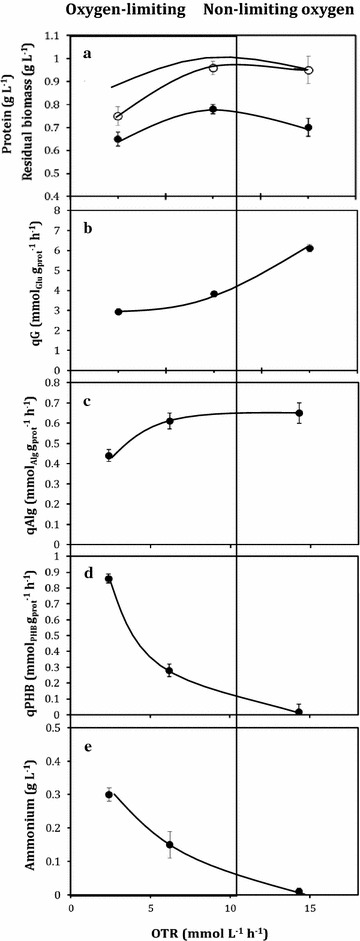
Cell growth as protein (**a**), specific sucrose uptake rate (**b**), specific alginate production rate (**c**), the specific P3HB production rate (**d**) and ammonium concentration (**e**) in *A. vinelandii* ATCC 9046, grown in continuous cultures (*D* = 0.08 h^−1^) under oxygen-limiting and non-limiting conditions. The error bars represent standard deviations

Cellular protein concentrations, residual biomass (CDW-P3HB), *q*_*G*_, *q*_*Alg*_ and *q*_*P3HB*_ of cells growing at the steady-state under oxygen-limiting and non-limiting conditions are shown in Fig. 2. Similar cellular protein concentrations were obtained under the different OTR conditions (around 0.71 ± 0.06 g L^−1^), as shown in Fig. 2a. Although the protein concentrations reached were very similar, the yields of protein with respect to the glucose consumed were different. The highest protein yield (0.15 g_protein_ g_glucose_^−1^) was obtained in the cultures developed under the lowest OTR (2.4 mmol L^−1^ h^−1^). In contrast, the protein yield was 46% lower under non-limiting oxygen conditions (14.3 mmol L^−1^ h^−1^) (Table 1). This behavior was also observed for the yields based on residual biomass (Table 1).

The residual glucose concentrations in the steady-state were 3.5 ± 0.3, 5.5 ± 0.35 and 1.3 ± 0.2 g L^−1^, for the OTR of 2.4, 6.2 and 14.3 mmol L^−1^ h^−1^, respectively. Under similar conditions, Díaz-Barrera et al. [29] reported an affinity constant (*Ks*) for sucrose of 0.1 g L^−1^. Therefore, the cultures were not limited by the carbon source. In addition, the ammonium concentration in the steady-state under oxygen-limiting conditions (2.4 and 6.2 mmol L^−1^ h^−1^) was higher (Fig. 2e) than the *K*_*S*_ described for ammonium in *A. vinelandii* (0.11 g L^−1^) [29]. Nevertheless, in cultures developed at 14.3 mmol L^−1^ h^−1^, the ammonium concentration was close to zero (Fig. 2e), suggesting that these cultures were probably fixing atmospheric nitrogen. As shown in Fig. 2b, when the OTR was higher (14.3 mmol L^−1^ h^−1^), the *q*_*G*_ increased (twofold) to 6.12 ± 0.35 mmol g^−1^ h^−1^, while the *q*_*G*_ at the lowest OTR condition was 2.9 ± 0.2 mmol g^−1^ h^−1^, showing a close correlation with the values of *qO*_*2*_ (Table 1).

The alginate and P3HB productions under the steady-state at the different OTR tested are shown in Table 1. Under oxygen-limiting conditions, the alginate production increased when the OTR was higher, from 0.7 ± 0.08 g L^−1^ at an OTR of 2.4 mmol L^−1^ h^−1^, to 1.18 ± 0.18 g L^−1^ when the cultures were conducted at 6.2 mmol L^−1^ h^−1^. A further increase in the OTR under non-limiting oxygen conditions (14.3 mmol L^−1^ h^−1^) did not increase alginate production (Table 1). Interestingly, the specific alginate production rate (*q*_*Alg*_) was similar (0.61 ± 0.04 mmol g_protein_^−1^ h^−1^) at the high OTR values (6.2 and 14.3); and this value was 35% higher than that achieved in cultures at 2.4 mmol L^−1^ h^−1^ (0.44 ± 0.03 mmol g_protein_^−1^ h^−1^).

The P3HB production in *A. vinelandii* has been reported to be accumulated mainly under oxygen-limiting conditions [2, 30, 31]. Consistently, in this study the P3HB production was negatively correlated by the increase in OTR, as shown in Table 1. The highest P3HB concentration (0.75 ± 0.04 g L^−1^) was obtained in cultures developed at 2.4 mmol L^−1^ h^−1^, whereas a value of 0.05 ± 0.002 g L^−1^ was obtained at the highest OTR (14.3 mmol L^−1^ h^−1^).

### Internal redox state, metabolic flux distribution and its corresponding gene expression

**Fig. 3:**
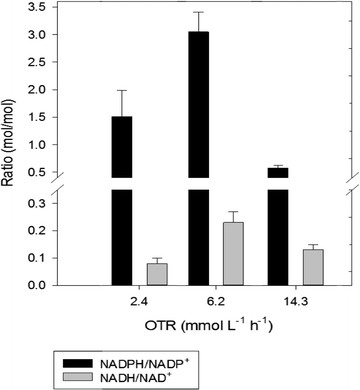
Cofactors NAD(P)H/NAD(P)^+^ ratio (mol/mol) in *A. vinelandii* ATCC 9046, grown in continuous cultures (*D* = 0.08 h^−1^) under oxygen-limiting and non-limiting conditions. The error bars represent standard deviations

The NADH/NAD^+^ ratio in cells growing under steady state conditions was significantly lower (4- to 20-fold) than the NADPH/NADP^+^ ratio under all of the conditions tested (Fig. 3). In the cultures under oxygen-limiting conditions, the intracellular NADH/NAD^+^ molar ratio, increased from 0.08 (at an OTR of 2.4 mmol L^−1^ h^−1^) to 0.23 mol/mol in cells grown at an OTR of 6.2 mmol L^−1^ h^−1^. Conversely, in cells grown at 14.3 mmol L^−1^ h^−1^, the NADH/NAD^+^ decreased by 35%, in relation to the 6.2 mmol L^−1^ h^−1^ condition. For the NADPH/NADP^+^ ratios, a similar response was obtained (Fig. 3). Under oxygen-limiting conditions, the ratio increased (83%) at 6.2 mmol L^−1^ h^−1^, with respect to the NADPH/NADP^+^ ratio achieved at 2.4 mmol L^−1^ h^−1^. For the steady-state under the non-limiting oxygen condition (14.3 mmol L^−1^ h^−1^), the NADPH ratio was 5.5- and 2.8-fold lower than that achieved in the cultures at 6.2 and 2.4 mmol L^−1^ h^−1^, respectively.

**Fig. 4:**
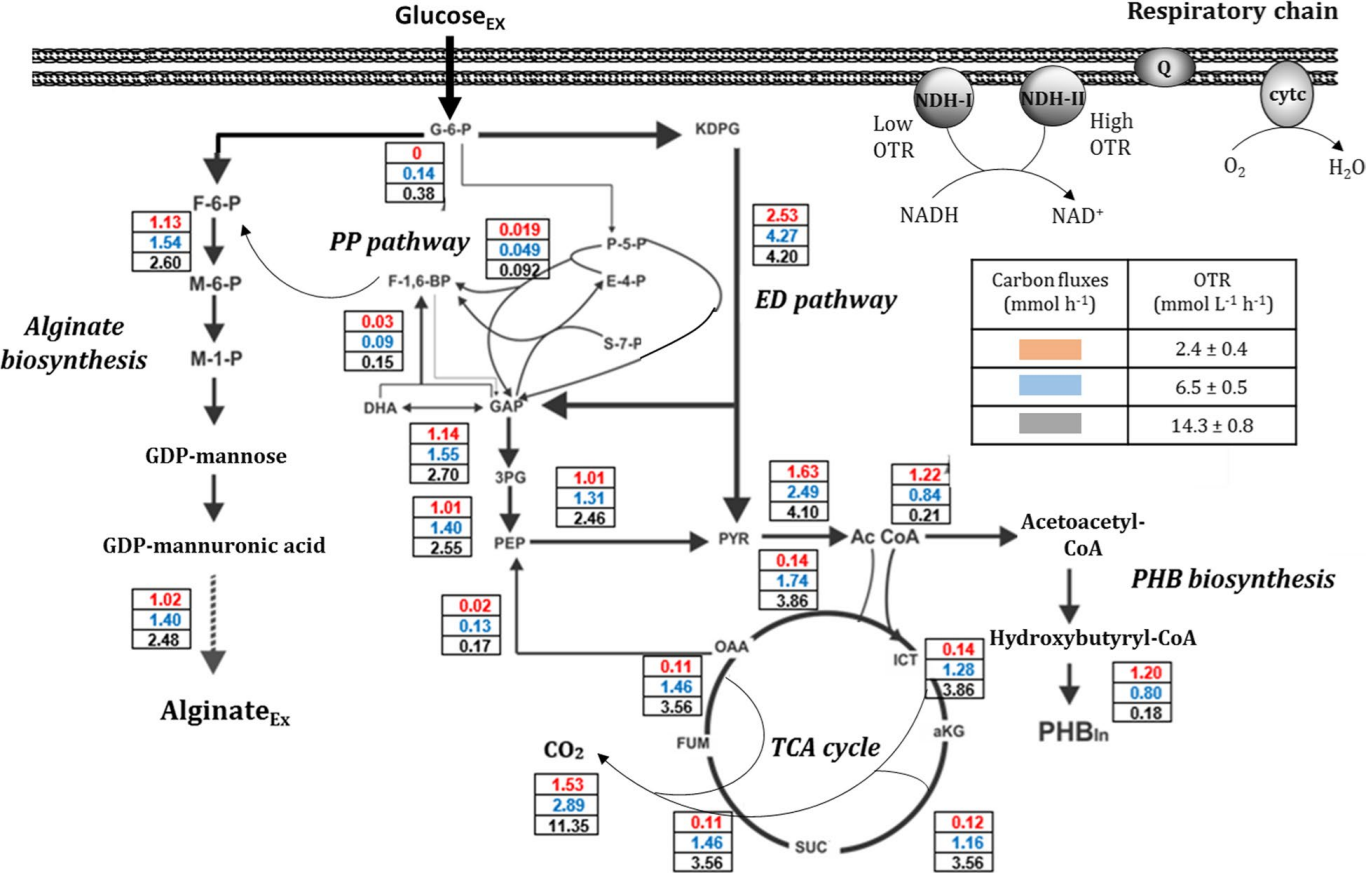
Metabolic pathways and carbon distribution in *A. vinelandii* ATCC 9046 grown in continuous cultures (*D* = 0.08 h^−1^) oxygen-limiting and non-limiting conditions. The relative carbon fluxes (mol%) are related to glucose uptake rates (100%)

The metabolic flux distribution of cells growing at steady-state under the three oxygenation conditions was evaluated (Fig. 4). In the cultures carried out at an OTR of 2.4 mmol L^−1^ h^−1^, the low consumption rate of the carbon source was reflected as a reduction in the relative flux of carbon towards central metabolism (ED, PP pathway and TCA cycle) compared to the relative fluxes achieved under OTR of 6.2 and 14.3 mmol L^−1^ h^−1^. For example, for the lower OTR, the relative fluxes through P3HB biosynthesis were sixfold higher than at the highest OTR (14.3 mmol L^−1^ h^−1^). In contrast, when *A. vinelandii* was grown under the non-limiting oxygen conditions (14.3 mmol L^−1^ h^−1^), fluxes towards the PP pathway and TCA cycle increased, being 4.84- and 30-fold higher than in the cultures conducted at the lowest OTR (2.3 mmol L^−1^ h^−1^). Such an increase was further reflected in a higher flux to CO_2_ production, which increased 7.4-fold at the highest OTR conditions. The distribution of the carbon usage in all of the evaluated conditions is shown in Table 2.

**Table 2:** % C-mole usage in *A. vinelandii* ATCC 9046, grown in continuous cultures (*D* = 0.08 h^−1^) under oxygen-limiting (2.4 and 6.2 mmol L^−1^ h^−1^) and non-limiting oxygen (14.3 mmol L^−1^ h^−1^) conditions

OTR_max_ (mmol L^−1^ h^−1^)	% C-mol biomass	% C-mol alginate	% C-mol P3HB	% C-mol CO_2_
2.4 ± 0.32	14.3	17.2	15.1	53.2
6.2 ± 0.57	11.7	18.4	4.3	65.6
14.3 ± 1.2	8.6	13.2	0.5	77.7

**Fig. 5:**
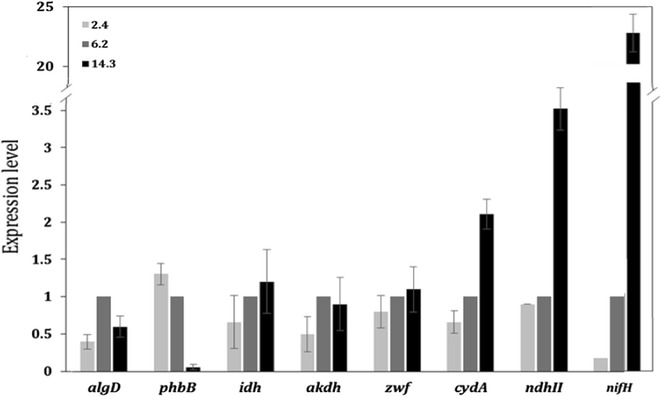
Expression level of *algD* (encoding alginate biosynthesis), *phbB* (encoding P3HB biosynthesis), *idh*, *akdh* and *zwf* (genes involved in central carbon metabolism), *cydA* and *ndhII* (genes involved in alternative respiratory chain) and *nifH* (encoding nitrogenase) in *A. vinelandii* ATCC 9046, grown in continuous cultures (*D* = 0.08 h^−1^) oxygen-limiting and non-limiting conditions

Figure 5 shows the influence of OTR on the transcription of genes coding for representative enzymes involved in central carbon metabolism (*akdh*, *idh* and *zwf*), the uncoupled respiratory chain (*ndhII* and *cydA*), alginate and P3HB biosynthesis (*algD* and *phbB,* respectively) and nitrogen fixation (*nifH*). No differences were observed in the expression levels of genes in central metabolism when comparing the cultures at OTRs of 2.4 and 14.3 mmol L^−1^ h^−1^, but their expression was around twofold lower and 1.3-fold higher, respectively, than the reference condition (6.2 mmol L^−1^ h^−1^). The expression level of *algD* was significantly lower (2.5-fold) in cultures developed at 2.4 mmol L^−1^ h^−1^, compared to the cultures developed at 6.2 mmol L^−1^ h^−1^ (reference condition), according to the decrease in alginate production, which was from 1.18 ± 0.18 to 0.7 ± 0.08 g L^−1^. The expression of this gene decreased 1.3-fold at the highest OTR (14.3 mmol L^−1^ h^−1^), although the alginate production was similar with respect to 6.2 mmol L^−1^ h^−1^ (Table 1). To determine whether there was a relationship between the P3HB production and relative expression, the level of *phbB* transcript was evaluated. Figure 5 shows that under non-limiting oxygen conditions, the *phbB* transcription levels decreased by about 1.4- and 13.3-fold for the OTR of 6.2 and 14.3 mmol L^−1^ h^−1^, respectively, in accordance with the decrease in P3HB production.

**Fig. 6:**
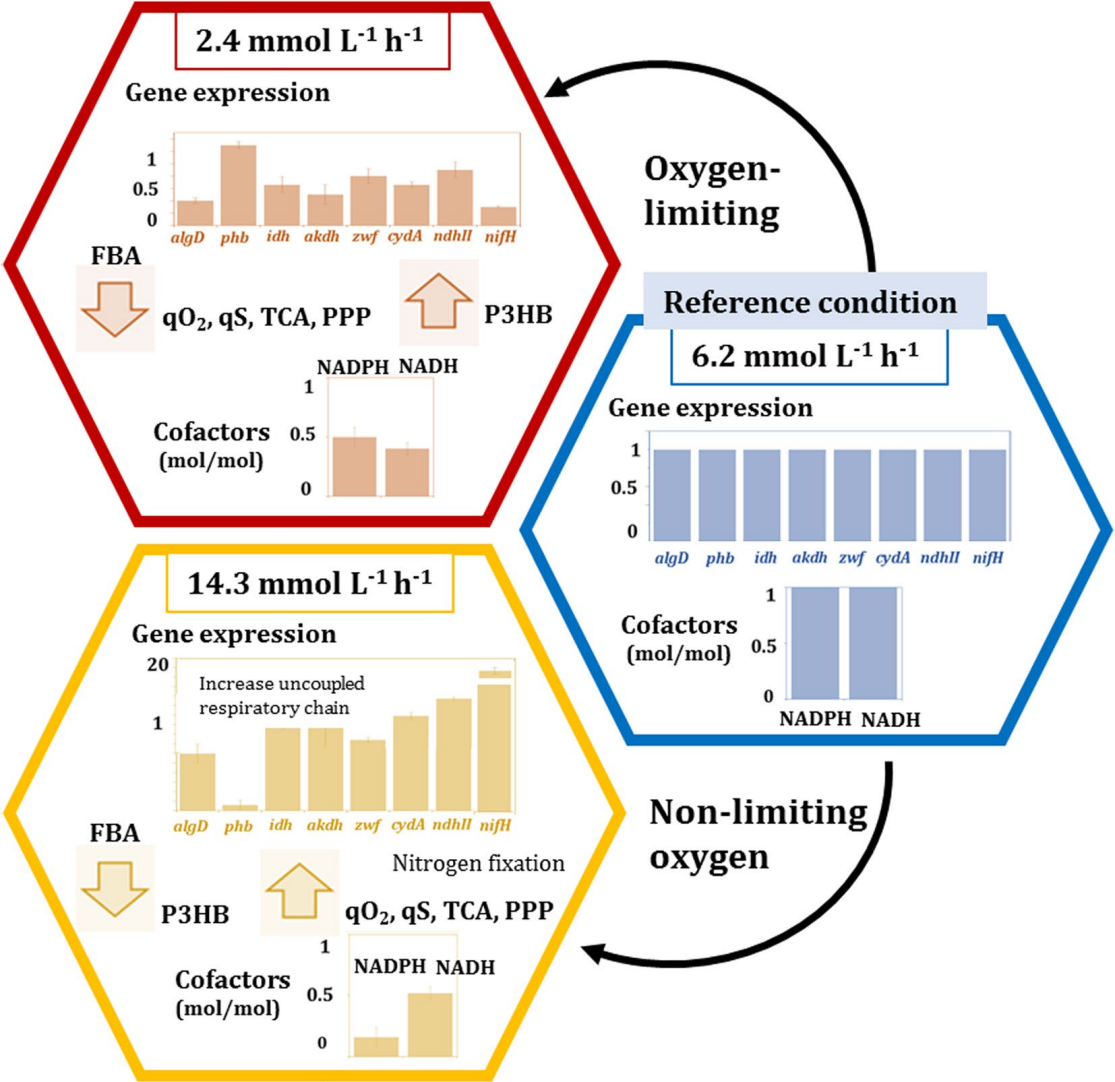
Schematic representation of different responses modulated by oxygen transfer rate and its relationship with metabolism of *A. vinelandii*. The hexagons represents the three different stages of oxygen consumption. The data was normalize with respect to the reference condition (6.2 mmol L^−1^ h^−1^)

In the cultures at 14.3 mmol L^−1^ h^−1^, where the protein yield was lower, the *q*_*G*_ was higher and the ammonium concentration was close to zero, the transcription levels of genes coding for components of the uncoupled respiratory chain (*cydA* and *ndhII*) were significantly induced with respect to the cultures developed under oxygen-limiting conditions. The *cydA* relative expression increased 30% when the OTR was 6.2 mmol L^−1^ h^−1^ with respect to 2.4 mmol L^−1^ h^−1^; however, an even higher induction was observed at 14.3 mmol L^−1^ h^−1^ (2.3-fold). For the *ndhII* gene, the relative expression was also considerably induced (3.5-fold) at the highest OTR of 14.3 mmol L^−1^ h^−1^ compared to the cultures developed at 6.2 mmol L^−1^ h^−1^ (reference condition). The results suggest that at the highest OTR (14.3 mmol L^−1^ h^−1^), the alternative respiratory chain involved in the respiratory protection of the nitrogenase is highly expressed, in agreement with the very low ammonium concentration (nitrogen limitation). Therefore, the expression of the nitrogenase was determined. The transcription level of *nifH,* the first gene of the *nifHDK* operon, containing structural genes of the nitrogenase complex, was induced 23-fold in the cultures at 14.3 mmol L^−1^ h^−1^ (where the oxygen consumption was increased), in comparison with 6.2 mmol L^−1^ h^−1^. This behavior is schematically represented in Fig. 6.

## Discussion

The results of the present study show, for the first time, that the OTR affects metabolic fluxes through the central carbon metabolism network and the NAD(P)H/NAD(P)^**+**^ ratios, thereby pointing at a relationship of the redox state with the alginate and P3HB production in chemostat cultures of *A. vinelandii*. Previous studies with this bacterium conducted in shaken flasks, under high and low aeration conditions, showed that changes in oxygen availability had a considerable impact on the growth profiles, the alginate and P3HB yields and metabolic fluxes [27]. However, shaken flasks are not the most suitable growth system for the evaluation of metabolic fluxes, because several non-controlled culture parameters (e.g. pH, DOT) and most of the relevant variables, substrate, product, or biomass concentrations continuously vary. Therefore, in this study, the continuous culture mode was chosen, because it allows the specific growth rate to be fixed, allowing a strict comparison among different conditions at the steady-state and also to evaluate the concentrations of NADH, NADPH and metabolic fluxes.

The cellular protein yield was twofold higher in those cultures conducted under oxygen limitation (2.4 and 6.2 mmol L^−1^ h^−1^) compared to the cultivations conducted at the highest (non-limiting) OTR (14.3 mmol L^−1^ h^−1^), although the highest *q*_*G*_ and *qO*_*2*_ were obtained under this last condition (Table 1). These differences might be related to the protein synthesis (under high OTR) of the alternative uncoupled respiratory chain of *A. vinelandii*, which has been reported to be active under high oxygen concentrations [32] and in diazotrophic conditions, allowing the oxygen sensitive nitrogenase complex to be protected. The results of the present study are in agreement with the functioning of the uncoupled respiratory chain of *A. vinelandii* [32], because under the highest OTR condition, some of its protein components, such as NADH oxidoreductase II and cytochrome bd oxidases were clearly induced (Fig. 5), probably as a response to the high oxygen concentration. This alternative respiratory chain has a high affinity for oxygen, but a low net ATP production [33]. These results, together with the analysis of carbon usage (% C-mol), suggest that the carbon source oxidized at higher oxygen consumption would be released as CO_2_ (Table 2), consume less energy for cell growth, in agreement with our results for *qO*_*2*_, Y_Protein/Glucose_ (Table 1) and *q*_*G*_ (Fig. 2b). On the other hand, this respiratory path is required under conditions of nitrogen fixation. This is in agreement with the requirement imposed by the NH_3_ limitation observed in the cultures under no oxygen-limited conditions at 14.3 mmol L^−1^ h^−1^ OTR (Fig. 2e) where the ammonium concentration was close to zero (Fig. 2e), suggesting that these cultures were probably fixing atmospheric nitrogen. Moreover, under this OTR condition, the transcription level of *nifH*, which is part of the *nifHDK* operon that encodes structural components of the nitrogenase system, was induced up to 60-fold with respect to its expression level at the lowest OTR condition. These results support the fact that *A. vinelandii* responds rapidly to nitrogen depletion under this condition by inducing the expression of nitrogenase; this is also related to the observed increase in the expression of the alternative uncoupled respiratory electron transport system that allows the nitrogenase to be protected under high oxygen transfer rates. As shown in Fig. 5, the relative transcription levels of *cydA* and *ndhII* (genes that encode the proteins cytochrome b and NADH dehydrogenase II of the uncoupled respiratory chain) increased up to threefold at 14.3 mmol L^−1^ h^−1^ compared to the cultivations developed at low OTR. It is also important to highlight that the enzymatic reduction of molecular nitrogen to ammonia requires high amounts of energy and reducing power (ATP and NADH) [34]. This explanation is supported by the fact that a higher relative metabolic flux through the TCA cycle and CO_2_ production was determined in the cultivations conducted at 14.3 mmol L^−1^ h^−1^ (Fig. 4).

In addition, as shown in Fig. 4, other metabolic pathways affected by the availability of oxygen were: the pyruvate and acetyl-CoA generation points, and the pentose phosphate (PP) pathway. Moreover, these differences were further reflected on the fluxes through the alginate and P3HB biosynthesis pathways. However, we found that the expression of genes encoding central metabolism (*idh, akdh* and *zwf*) were similar under all of the conditions evaluated (Fig. 5). These results were inconsistent with the differences in carbon flux towards the central metabolism (PP pathway and TCA cycle). In the cultures at higher oxygen consumption (14.3 mmol L^−1^ h^−1^), the relative flux through the PP pathway was nearly 1.8- and 4.8-fold higher than the flux achieved in cultures at 6.2 and 2.4 mmol L^−1^ h^−1^, respectively. A similar increase was observed in the metabolic fluxes towards alginate production when this bacterium was grown at high OTR (6.2 and 14.3 mmol L^−1^ h^−1^) reaching the highest fluxes at 14.3 mmol L^−1^ h^−1^. Previous studies have shown that when *A. vinelandii* is grown in continuous cultures, without DOT control (oxygen-limited), the alginate concentration can also be increased by increasing *qO*_*2*_ [29, 35]. When the OTR, and therefore, the *qO*_*2*_ increased, the carbon fluxes through alginate biosynthesis pathway also increased, from 1.1 at 2.4 mmol L^−1^ h^−1^ to 2.6 when the OTR was 14.3 mmol L^−1^ h^−1^. These changes at the metabolic flux level are associated with the increase in *q*_*Alg*_ values, which increased from 0.45 mmol g^−1^ h^−1^ in cultures developed at the lowest OTR, to 0.6 mmol g^−1^ h^−1^ when the OTR was 14.3 mmol L^−1^ h^−1^. However, this value is the same in the cultures developed at 6.2 mmol L^−1^ h^−1^ (Fig. 2c). These results suggest that changes in the cellular respiration (in the range from 6.2 to 14.3 mmol L^−1^ h^−1^) did not affect alginate biosynthesis. In contrast, Sabra et al. [34] reported that the alginate could serve as a barrier to protect the nitrogenase system at high OTR and under diazotrophic conditions. One possible explanation for this behavior could be that, under non-limiting oxygen conditions (14.3 mmol L^−1^ h^−1^), the energy demand (in the form of ATP) increased, for nitrogen fixation purposes, as well for the mechanism known as respiratory protection instead of being used to increase alginate biosynthesis [36]. The induction of *algD* gene transcription is a key point in regulation of the alginate synthesis pathway and is mediated by alginate switching and regulatory genes. In this study, we found that the transcription of the *algD* gene was higher at 6.2 mmol L^−1^ h^−1^ (Fig. 5) and these results were consistent with the higher alginate production under this condition. However, the alginate production was similar in cultures at higher oxygen consumption (14.3 mmol L^−1^ h^−1^) when the *algD* transcription level decreased.

On the other hand, the carbon fluxes towards P3HB production were considerably higher (sixfold) at the lowest OTR condition, in contrast to the flux through this pathway observed for those cultivations conducted at 14.3 mmol L^−1^ h^−1^. These findings correlate with the *q*_*P3HB*_, which was 49-fold higher in those cultures conducted with an OTR of 2.4 mmol L^−1^ h^−1^ compared to those developed at the highest OTR. Our results are consistent with previous studies showing that P3HB biosynthesis in *A. vinelandii* is a response to oxygen limitation and carbon excess [27]. As shown in Fig. 5, the *phbB* transcription levels increased at 2.4 mmol L^−1^ h^−1^ in correspondence with the higher production of P3HB observed under that condition. Several authors have proposed that NADPH concentration increases under oxygen-limiting conditions, thereby inhibiting the activities of the tricarboxylic acid (TCA) cycle enzymes, citrate synthase and isocitrate dehydrogenase. Subsequently, the inhibition of these TCA enzymes would cause an increase in the acetyl-CoA available to flux through the P3HB synthesis pathway [2, 37]. On the other hand, as shown schematically in Fig. 6, we observed a slight decrease in gene expression levels of the TCA enzymes ICTDH and aKGDH, in those cultivations conducted at the lowest OTR, as compared to these genes transcription levels at high OTR in agreement with the fluxes discussed.

In the present study, we observed that the internal redox state (reflected by the NADH/NAD^+^ and NADPH/NADP^+^ ratios) was affected by changes in the OTR (Fig. 3). Additionally, it was observed that independently of the OTR tested, the NADH/NAD^+^ ratio was always significantly lower than the NADPH/NADP^+^ ratio. Under oxygen-limiting conditions (OTR = of 2.4 mmol L^−1^ h^−1^), the NADPH/NADP ratio was half of that obtained in the cultures performed at 6.2 mmol L^−1^ h^−1^. There is probably more NADPH regenerated at the intermediate oxygen-limiting condition than at extreme oxygen limitation, as we can see higher flux through the PP pathway (an important NADPH generation pathway) was higher (2.5-fold) when the OTR was 6.2 mmol L^−1^ h^−1^.

It is important to point out that the NADH cofactor is mainly generated in the central metabolic pathways [Entner–Doudoroff (ED) pathway, TCA cycle and alginate biosynthesis], being consumed mainly in the respiratory chains to produce energy in the form of ATP. Although the values of the NADH/NAD^+^ ratios achieved under the different conditions changed slightly with OTR (Fig. 3), they were always low. This could be due to fact that the NADH is rapidly consumed by the respiratory chains, and a high proportion of the oxidized form NAD^+^ has to be preserved in order to favor oxidation of the carbon source; therefore, this intracellular ratio is low. Nevertheless, there were significant differences in the NADH/NAD ratios among the different cultivation conditions evaluated. It is known, that when the oxygen consumption rate increases, the total dinucleotide pool generated during the oxidation of glucose is consumed by the first protein complex of the respiratory chain [38]. Therefore, the intracellular ratio of this cofactor is expected to decrease as the OTR increases. It is important to point out that at an OTR of 6.2 mmol L^−1^ h^−1^, the highest NADH/NAD ratio (0.23) was achieved, which is in agreement with an increase in the carbon fluxes through the alginate biosynthesis and TCA cycle, both of which are NADH regeneration pathways (Fig. 4). Therefore, it is possible that under this condition, alginate biosynthesis contributes to increase the NADH availability. On the other hand, when the OTR increased up to 14.3 mmol L^−1^ h^−1^, the *A. vinelandii* cells were not oxygen-limiting, but were exposed to nitrogen limitation conditions. Under these condition, although the carbon fluxes through the TCA cycle, and therefore for NADH production, were higher than under the other conditions evaluated, the electrons of cofactors (NADH and NADPH) could be consumed faster in the respiratory chains of *A. vinelandii* (the coupled and uncoupled) as part of the protection of the nitrogenase system under high oxygen concentrations [36], and it was reflected as a decrease in the NADH/NAD^+^ ratio (Fig. 6).

In *A. vinelandii*, the main NADPH producing pathways are the ED pathway, the isocitrate dehydrogenase step of the TCA cycle and PPP; these pathways allow a high NADPH/NADP^+^ ratio to be achieved [39]. The reduced form NADPH has a significant contribution during reductive biosynthetic reactions (fatty acids, amino acids and P3HB), and when *A. vinelandii* is exposed to high oxygen concentrations and nitrogen fixation, this cofactor could be directed through the uncoupled respiratory chain by NADH dehydrogenase II [32]. The above fact suggests that, the lower NADPH/NADP^+^ ratio observed in those cultivations conducted at the highest OTR could be due to an increase in NADH dehydrogenase II activity.

## Conclusions

The NADH/NAD^+^ and NADPH/NADP^+^ cofactor pairs are involved in many biochemical reactions. In the case of *A. vinelandii*, it has been shown that changes in the oxygen availability have an important effect on the metabolic fluxes. Our results show that at the lowest OTR (2.4 mmol L^−1^ h^−1^), the NAD(P)H levels were lower than those achieved at 6.2 mmol L^−1^ h^−1^, probably due to a decrease in the carbon fluxes towards the central metabolism (PP pathway an TCA cycle) and to an increase in P3HB biosynthesis. However, the decrease of these reduced cofactor pairs under non-limiting oxygen conditions (14.3 mmol L^−1^ h^−1^) compared to the condition at 6.2 mmol L^−1^ h^−1^, might be due to the process of nitrogen fixation and the respiratory protection of *A. vinelandii* that this work shows being induced at high oxygen availability, which was associated with the nitrogen limitation that occurs under this condition.

## Methods

### Bacterial strain and culture medium

*Azotobacter vinelandii* wild-type strain ATCC 9046 was used. The strain was conserved at 29 °C in Burk’s nitrogen-free salts supplemented with 20 g L^−1^ of glucose [31]. The composition of the culture medium was as follows (in g L^−1^): K_2_HPO_4_ 0.66, KH_2_PO_4_ 0.16, MOPS 1.42, CaSO_4_·2H_2_O 0.05, NaCl 0.2, MgSO_4_ 0.2, NaMoO_4_·2H_2_O 0.0029, FeSO_4_·7H_2_O 0.027 and 0.8 (NH_4_)_2_SO_4_. The glucose concentration used for chemostat stage cultivation, was of 10 g L^−1^. The initial pH was adjusted to 7.2 using 2 N NaOH.

### Chemostat cultures

Continuous cultures were carried out in a 3 L Applikon bioreactor (Schiedam, Netherlands) with 2 L working volume. The bioreactor, was equipped with two Rushton turbines and aerated at 2 L min^−1^ (1.0 vvm). The cultivations were conducted at 300, 500 and 700 rpm and the pH was kept constant at 7.2 and it was controlled by the addition of 2 N NaOH. Temperature was maintained at 29 °C. The DOT was measured using a polarographic oxygen probe (Applikon Schiedam, Netherlands). The continuous cultures were operated a dilution rate of 0.08 h^−1^, this value corresponded to 80% of *μ*_max_ at 300 rpm. The values of *μ*_max_ were determined in batch cultivations at different agitation rates using the same culture conditions (pH 7.2, 29 °C, 1.0 vvm). The steady-state condition was achieved after three residence times, when the protein and the glucose concentration remained constant (≤ 5% variation). Samples of cultures (50 mL) were withdrawn from the bioreactor for analytical measurements. All the experiments were conducted by triplicate and the mean value of these results was calculated and reported.

### Analytical determinations

#### Cellular protein, glucose and ammonium concentration assessments

Microbial growth was evaluated through protein measurements using the Lowry method [40], with bovine serum albumin as standard, the residual biomass was estimated by subtracting the P3HB content from the total biomass. Glucose concentration was determined by the dinitrosalicylic acid (DNS) reagent [41]. Ammonium concentration was measured by the phenol-hypochlorite method, as described by Kaplan [42].

#### Poly(3-hydroxybutyrate) (P3HB) and alginate quantification

P3HB content was quantified by HPLC, after its conversion into crotonic acid. Firstly, the biomass was dried under vacuum at 60 °C. For each sample, 3 mg of biomass was weighed in a 1.5 mL Eppendorf tube; 1 mL of H_2_SO_4_ was added, and the sample was heated at 90 °C for 1 h. Subsequently, the sample was cooled at room temperature and diluted with Milli-Q water to concentrations within the range of the calibration curve. The P3HB quantification was performed using an HPLC system with an Aminex HPX-87H column (300 × 7.8 mm) (Bio-Rad, Hercules, CA, USA) at 50 °C and using H_2_SO_4_ (7 mM) as the eluent with a flow rate of 0.65 ml min^−1^. Crotonic acid was quantified using UV absorption at 220 nm. Commercial P3HB (Sigma-Aldrich), treated identically as the samples was used as standard. Alginate concentration was quantified gravimetrically by precipitation with 3 volumes of ice propanol. The precipitate was filtered (0.22 μm Millipore filters) and dried at 85 °C to constant weight [43].

#### Determination of the OTR/OUR and the specific oxygen uptake rate

Gas analysis was performed by online measurements of O_2_ and CO_2_ in the exit gas and compared with measurements taken of the inlet gas with a gas analyzer (Teledyne Instruments, USA). The following equation was applied to calculate OTR from the exit gas [44]:

1 $$OTR = \frac{{V_{G} }}{{V_{L} V_{N} }}\left( {X_{in} - X_{out} } \right)$$

V_G_ is the gas inlet flow rate (L h^−1^), V_L_ the fermentation working volume (L), V_N_ the molar volume (L mol^−1^), and X_in_ and X_out_ the mole fractions at the gas inlet and outlet, respectively. For chemostat cultures at constant DOT and for conditions of steady state, the OTR (mmol L^−1^ h^−1^) is equal to the oxygen uptake rate (OUR) [45], and the equation used for determination of specific the oxygen uptake rate (*qO*_*2*_) was as follows:

2 $$qO_{2} = \frac{{OUR_{\text{max} } }}{{X_{\text{max} } }}$$

where OUR_max_ (mmol L^−1^ h^−1^) was the maximum oxygen uptake rate and X_max_ (g L^−1^) was the maximum protein concentration.

#### Measurements of the intracellular cofactors concentrations

Amounts of NAD^+^, NADH, NADP^+^, and NADPH were quantified by enzymatic methods [46]. These cofactors, were extracted and assayed using the EnzyChrom™ assay kit following the supplier’s instructions (BioAssay Systems, Hayward, CA, USA). 12.6 mg of wet cells from cultures were immediately received in methanol (70% v/v) at − 50 °C for a rapid inactivation of the cellular metabolism [23]. The cell pellet was washed with cold PBS and resuspended with base or acid buffer (BioAssay Systems, Hayward, CA, USA) to extract the reduced or oxidized pyridine nucleotides [46]. The assays utilized glucose-6-phosphate dehydrogenase and lactate dehydrogenase for NAD(H) and NADP(H) quantification respectively, at 565 nm (BioAssay Systems, Hayward, CA, USA).

### Flux balance analysis

The intracellular fluxes were determined by flux balance analysis (FBA), using substrate uptake rates and product formation rates by applying metabolite balancing, which is based on the stoichiometry of all reactions in the metabolic network [31]. The model developed for *A. vinelandii* included the metabolic network described in Table 3, based on the Entner–Doudoroff (ED) pathway, the pentose phosphate (PP) pathway, the TCA cycle, as well as the alginate and P3HB biosynthetic pathways. FBA simulations were performed using the OptFlux 3.3, an open-source and modular software for in silico metabolic engineering [47]. Maximizing cellular growth rate was used as the objective function for all FBA simulations.

**Table 3:** The components of the metabolic network used for flux calculation were as follows

v1a	GLC	→	G6P
v1b	G6P	→	F6P
v1c	F6P	→	GAP + DHA
v2	DHA	→	GAP
v3	GAP	→	c3PG
v4	c3PG	→	PEP
v5	PEP	→	PYR
v6	PYR	→	ACCOA + CO_2_
v7	PYR + CO_2_	→	OAA
v8	OAA	→	PEP + CO_2_
v9	G6P	→	P5P + CO_2_
v10	P5P	→	GAP + S7P
v10r	GAP + S7P	→	P5P
v11	GAP + S7P	→	cE4P + F6P
v11r	cE4P + F6P	→	GAP + S7P
v12	cE4P + P5P	→	GAP + F6P
v12r	GAP + F6P	→	cE4P + P5P
v13	OAA + ACCOA	→	ICT
v14	ICT	→	CO_2_ + aKG
v15	aKG	→	SUC + CO_2_
v16	SUC	→	FUM
v16r	FUM	→	SUC
v17	FUM	→	OAA
v18	ICT	→	SUC + GOX
v19	ACCOA + GOX	→	OAA
v20	G6P	→	KDPG
v21	KDPG	→	PYR + GAP
v22	F6P	→	MAN6P
v1ext	MAN6P	→	Alginate
v2in	ACCOA	→	P3HB_bio
vB_G6P	G6P	→	G6P_bio
vB_F6P	F6P	→	F6P_bio
vB_GAP	GAP	→	GAP_bio
vB_c3PG	c3PG	→	c3PG_bio
vB_PEP	PEP	→	PEP_bio
vB_PYR	PYR	→	PYR_bio
vB_ACCOA	ACCOA	→	ACCOA_bio
vB_OAA	OAA	→	OAA_bio
vB_AKG	aKG	→	aKG_bio
vB_P5P	P5P	→	P5P_bio
vB_cE4P	cE4P	→	cE4P_bio
vB_Biomass	0.0228 * c3PG_bio + 0.0024 * PEP_bio + 0.0012 * cE4P_bio + 7.0E−4 * G6P_bio + 0.0194 * OAA_bio + 0.2768 * ACCOA_bio + 0.0062 * P5P_bio + 0.0512 * PYR_bio + 0.0267 * aKG_bio	→	Biomass
vB_Biomass_ex	Biomass	→	Biomass_ex
vGlcinp	GLC	↔	GLC_ex
vAlgex	Alginate	↔	Alginate_ex
vexCO_2_	CO_2_	→	CO2_ex

### Quantitative RT-PCR

Expression of *algD, phbB, cydA, ndhII* and *nifH* were measured by quantitative RT-PCR (*qRT*-*PCR*), as previously reported [48]. Total RNA extraction was performed as previously reported [49]. To eliminate genomic DNA, RNA was treated with DNase (DNA-free™, Ambion) and its concentration measured by 260/280 nm ratio absorbance. The cDNA was synthesized using 500 ng of DNase-treated total RNA, the Revert Aid™ H First Strand cDNA Synthesis kit (Fermentas), and a mixture of the specific DNA primers. The sequences of the primers used are listed in Table 4. The cDNA obtained was used as template for Real-Time PCR assays. The level of *gyrA* was used as an internal control to normalize the results. All assays were performed in triplicate. The data were analyzed by the 2^−Δ,ΔCT^ method reported by Livak and Schmittgen  [50]. The data are reported as relative expression levels by comparing expression levels in cultures developed at 6.2 and 14.3 mmol L^−1^ h^−1^, to the expression levels of reference value (culture developed at 2.4 mmol L^−1^ h^−1^).

**Table 4:** Primers used for the quantitative RT-PCR

Gene	Forward primers (5′–3′)	Reverse primers (5′–3′)
*gyrA*	CCAGCAAGGGCAAGGTCTA	TCGTCCAGCGGCAACAGGT
*algD*	ACGTGGTCATTCCGATTCTC	GCGGGAAGTTGTAGTCCTTG
*phbB*	GGAAAACTTCACCGTGATCG	CGTGTTCGCAGACTTCAAA
*idh*	CCCCTGTCCGTCAAGAACTA	GCCGTAGAAATCACCGTTGT
*akdh*	TCAAGCATGTGGTCTGGTGT	GGAACAGCTCCTTCTTGTGC
*zwf*	ATGATCCAGAACCACCTGCT	CCTTGAGCACCTTGACCTTC
*cydA*	CCTCCGAAGAGCAGATCAAG	GCAGACAAACAGGATGAGCA
*ndhII*	GTGCGCAAGGTACCGTAGAG	GTAACCTGATGGGCAACCTG
*nifH*	TCGACAACAAACTGCTGGTC	ACGATGGATTCGTCTTCGAC

### Mathematical expressions

The specific glucose uptake rate (*q*_*G*_) and specific alginate and P3HB production rate (*q*_*P*_) in steady-state conditions were calculated as follows:

#### Specific glucose uptake rate

$$q_{G} = \frac{{D\left( {G_{0} - G} \right)}}{X}$$

#### Specific alginate or P3HB production rate

$$q_{p} = \frac{DP}{X}$$

where *D* (h^−1^) is the dilution rate, *P* (g L^−1^) is the alginate or P3HB concentration, *X* (g L^−1^) is a protein concentration, *Go* (g L^−1^) is the glucose concentration in the feed medium, and *G* (g L^−1^) is the residual glucose concentration.

### Carbon usage determination

The % C-mol was calculated as the ratio of the C-mol of biomass and products and the C-mol of glucose assuming that the carbon source consumed was 100%. The C-mol of biomass results from the biomass elemental composition described as CH_2_O_0.52_N_0.16_ (24.6 g C-mol^−1^). For alginate and PHB, the monomeric composition was considered: hydroxybutyric acid for PHB and mannuronic acid and acetyl groups for alginate. The following values were used: 24.6 g CDW = 1C-mol for % C-mol biomass; 26.0 g hydroxybutyric acid = 1C-mol for % C-molPHB; 32.3 g mannuronic = 1C-mol for % C-molAlg; 21.5 g acetyl = 1C-mol for % C-molAcetyl. The % C-mol of CO2 was estimated as the difference between the 100% C-mol and the  % C-mol used for the biomass and products.
